# Diabetes aggravates renal ischemia and reperfusion injury in rats by exacerbating oxidative stress, inflammation, and apoptosis

**DOI:** 10.1080/0886022X.2019.1643737

**Published:** 2019-08-23

**Authors:** Dao-Jing Gong, Lei Wang, Yuan-Yuan Yang, Jian-Jian Zhang, Xiu-Heng Liu

**Affiliations:** Department of Urology, Renmin Hospital of Wuhan University, Wuhan, Hubei, P.R. China

**Keywords:** Diabetes mellitus, ischemia-reperfusion, oxidative stress, inflammation, acute kidney injury, apoptosis

## Abstract

Diabetic patients are more susceptible to renal ischemia/reperfusion (I/R) injury (RI/RI) and have a poor prognosis, but the underlying mechanism remains unclear. The present study aimed to examine whether diabetes could worsen acute kidney injury induced by I/R in rats and clarify its mechanism. Control and streptozotocin-induced diabetic rats were subjected to 45 min renal pedicle occlusion followed by 24 h reperfusion. Tert-butylhydroquinone (TBHQ, 16.7 mg/kg) was administrated intraperitoneally 3 times at intervals of 8 h before ischemia. Serum and kidneys were harvested after reperfusion to evaluate renal function and histological injury. Enzyme-linked immunosorbent assays were used to test pro-inflammatory cytokines. Terminal deoxynucleotidyl-transferase-mediated dUTP nick-end labeling assays were used to detect apoptotic cells, and western blotting was performed to determine the expression of B-cell lymphoma-2 (Bcl-2), Bcl-2-associated X protein (Bax), and cleaved caspase-3, as well as oxidative stress and inflammation-related proteins, such as nuclear factor-erythroid 2-related factor 2 (Nrf2), heme oxygenase-1 (HO-1), Toll-like receptor 4 (TLR4), and nuclear factor-κB (NF-κB). Compared with control animals, diabetic rats undergoing I/R exhibited more severe tubular damage and renal dysfunction. Diabetes exacerbated oxidative stress, the inflammatory response, and apoptosis after renal I/R by enhancing TLR4/NF-κB signaling and blocking the Nrf2/HO-1 pathway. RI/RI in diabetic rats was attenuated by pretreatment with TBHQ (a Nrf2 agonist), which exerted anti-inflammatory and anti-apoptotic properties by inhibiting NF-κB signaling. These findings indicate that hyperglycemia exacerbates RI/RI by intensifying oxidative stress, inflammation, and apoptosis. Antioxidant pretreatment may alleviate RI/RI in diabetic patients.

## Introduction

Ischemia/reperfusion (I/R) injury refers to tissue damage caused by blood returning to tissue after a period of ischemia. Renal I/R injury (RI/RI) is a major cause of acute postoperative renal dysfunction that leads to detrimental outcomes in hospitalized patients. It is common in renal surgeries including kidney transplantation and partial nephrectomy, resulting in serious complications such as delayed graft function, graft rejection, and acute kidney injury (AKI) [1,2]. The mechanisms contributing to I/R injury pathogenesis involve multiple highly integrated pathophysiological processes, including calcium overload, oxidative stress, endoplasmic reticulum stress, mitochondrial dysfunction, apoptosis, and inflammation [3]. Notably, oxidative stress and inflammation are pivotal to initiate RI/RI. During reperfusion, excessive reactive oxygen species (ROS) are produced due to abrupt regaining of oxygen and cause serious damage to cell proteins, lipids, and DNA, leading to cell apoptosis and necrosis [4].

Diabetes mellitus is a common disease in surgical patients and is considered a major risk factor for a variety of surgical complications [5–7]. In particular, diabetes is linked to poor prognosis following I/R injury. As previously reported, I/R damage of the heart, liver, and nervous tissues tends to be more serious in diabetic patients [8–10]. Hyperglycemia is the defining characteristic of diabetes and can elevate the basal level of ROS in cardiomyocytes, hepatocytes, and endothelial cells, causing a state of chronic oxidative stress [9,11,12]. An increase in baseline ROS production is a key factor that aggravates cardiac I/R injury [13]. Furthermore, diabetes also blunts the cardioprotective effects of sevoflurane postconditioning against myocardial I/R injury by impairing cardiac antioxidant capacity [14].

Chronic kidney disease (CKD) is characterized by a gradual loss of kidney function over a period of months or years. The main causes of CKD are diabetes, high blood pressure, and glomerulonephritis. Diabetes-induced kidney damage, also known as diabetic nephropathy, can result in renal impairment, urinary protein loss, and ultimately CKD [15]. Less attention has been paid to AKI susceptibility in CKD, but some studies have reported RI/RI susceptibility in CKD [16–18]. Evidence for a correlation between diabetes and worse outcome following RI/RI is accumulating [19–21]. Several diabetic rat models demonstrated roles of hyperglycemia, inflammation, and apoptosis in AKI susceptibility [22–24]. However, how diabetes contributes to negative renal surgery outcome remains unknown.

Given the rapidly rising incidence of diabetes [25], more research is required to understand RI/RI in diabetic patients. Here, we established an RI/RI model in streptozotocin (STZ)-induced diabetic rats to investigate the hypotheses: (1) Hyperglycemia aggravates RI/RI through increasing oxidative stress, inflammation, and apoptosis; (2) Antioxidant pretreatment may enhance renal antioxidant capacity and alleviate RI/RI in the context of diabetes.

## Material and methods

### Animal model of diabetes

Adult male Sprague Dawley rats weighing 200–220 g were purchased from Beijing Vital River Laboratory Animal Technology Co., Ltd. (Beijing, China). This study was approved by the Committee of the Animal Experimental Center of Wuhan University, and the procedures were conducted in accordance with the Guidelines of the Care and Use of Laboratory Animals. All rats were raised under specific pathogen-free conditions (23 ± 3°C; relative humidity 40–70%) under a 12-h light/dark cycle. All rats were adaptively fed with free access to water and standard rat chow for 1 week before the experiment. To induce RI/RI in a high glucose environment, we randomly divided the rats into four groups (*n* = 8 per group): (1) normal sham (NS), (2) normal I/R (NI/R), (3) diabetic sham (DS), and (4) diabetic I/R (DI/R). To further demonstrate the effect of tert-butylhydroquinone (TBHQ) on RI/RI in a high glucose condition, we randomly distributed the confirmed diabetic rats into three groups (*n* = 8 per group): (1) DS, (2) DI/R, and (3) DI/R + TBHQ. After an overnight fast, rats in the diabetic groups were injected intraperitoneally (i.p.) with a single dose of 65 mg/kg STZ (Sigma-Aldrich, St. Louis, MO, USA) dissolved in citrate buffer (0.1 mM, pH 4.5), and rats in the control groups received an equal volume of citrate buffer. Three days after STZ administration, blood was obtained from the tail vein to measure blood glucose level with a glucometer (Terumo, Tokyo, Japan). Animals with random plasma glucose >16.7 mM for three consecutive readings were considered diabetic [26]; these animals were supplied with sufficient normal diet and water *ad libitum* and housed for another 8 weeks before renal I/R.

### Renal I/R injury

Body weight and blood glucose were measured before surgery. Rats were generally anesthetized by continuous spontaneous inhalation of 2–5% isoflurane (cat. no. 045727; Lunan Better Pharmaceutical Co., Ltd., Shandong, China) through a portable anesthesia machine (cat. no. AS-01–0007; Summit Anesthesia Solutions, Bend, OR, USA). Following midline laparotomy, a right nephrectomy was performed followed by left renal pedicle clamping for 45 min with a non-traumatic vascular clamp. Ischemia was successfully induced when kidney color changed from red to pale. After clamp removal, blood flow restoration was considered successful when the kidney color changed from gray to red, and then, the abdomen was closed. Sham-operated animals were subjected to the same protocol without renal pedicle clamping. After the operation, rats were placed on a 37°C heated pad until consciousness was regained. All animals were sacrificed by withdrawal of blood from the inferior vena cava after 24-h reperfusion. Blood samples were collected for renal function tests. The left kidneys were removed and sliced coronally into two pieces for further analysis. One piece was fixed in 10% formalin and embedded in paraffin, and the other was immediately stored at –80°C.

### TBHQ treatment

A total of 100 mg TBHQ (cat. no. 112941, Sigma-Aldrich) was dissolved in dimethyl sulfoxide (DMSO) and then diluted by phosphate-buffered saline (PBS) to a final concentration of 5 mg/ml TBHQ, 1% DMSO in PBS. About 16.7 mg/kg TBHQ was administrated i.p. 3 times at intervals of 8 h prior to renal I/R [27]. The vehicle solution was the same concentration of DMSO in PBS. The injection solution was prepared fresh every day.

### Histopathological examination

After tissue fixation and embedding, 4-μm thick sections were obtained for hematoxylin and eosin (H&E) staining. Morphological changes were assessed by two experienced renal pathologists unaware of the assigned treatments. The degree of I/R-induced renal lesion was graded from 0 to 4 histologically according to the following criteria [28]: 0, no tubular necrosis; 1, necrosis of individual proximal convoluted tubule cells; 2, necrosis involving all cells in adjacent proximal convoluted tubules with survival of surrounding renal tubules; 3, necrosis limited to the distal third of the proximal convoluted tubule with the inner cortex affected; 4, necrosis spreading to all three proximal convoluted tubule segments. Ten different fields in each section were randomly selected to calculate the tubular injury score using a light microscope (magnification, ×400, Olympus Corporation, Tokyo, Japan).

### Assessment of renal function

Whole blood was centrifuged at 2000 × g for 15 min at 4°C to obtain serum. Blood urea nitrogen (BUN) and serum creatinine (SCr) were determined using a Hitachi 7060 automatic biochemistry analyzer (Hitachi, Ltd., Tokyo, Japan).

### Malondialdehyde and superoxide dismutase measurement

Superoxide dismutase (SOD) is a significant enzyme in oxidative stress, and malondialdehyde (MDA) is a terminal product of lipid peroxidation. After washing with precooled PBS, renal tissues were cut into fragments and homogenized on ice with a glass homogenizer. The homogenates were centrifuged at 12,000 × g for 10 min, and the supernatants were collected. MDA levels and SOD activity in kidney tissues were measured using commercial assay kits (Jiancheng Bioengineering Institute, Nanjing, China) following the manufacturer′s protocols.

### Enzyme-linked immunosorbent assay

Levels of tumor necrosis factor (TNF)-α and interleukin (IL)-1β in homogenized renal tissues were detected using rat-specific enzyme-linked immunosorbent assay (ELISA) kits according to the manufacturer′s instructions (Elabscience Biotechnology Co., Ltd, Wuhan, China). The optical density (OD) values of TNF-α and IL-1β were measured with an automatic microplate reader (Thermo Fisher Scientific, Waltham, MA, USA) at 450 nm. ELISA standard curves were established according to the concentrations and their corresponding OD values.

### Terminal deoxynucleotidyl-transferase-mediated dUTP nick-end labeling

Renal apoptosis was detected with an In Situ Cell Death Detection Kit according to the manufacturer′s instruction (cat. no. 12156792910; Roche Diagnostics GmbH, Mannheim, Germany). Briefly, 4-μm-thick paraffin-embedded sections were first deparaffinized in graded xylene-alcohol solutions and treated with proteinase K solution (20 μg/ml) at 37°C for 15 min. Subsequently, sections were washed with PBS 3 times and incubated with the terminal deoxynucleotidyl-transferase-mediated dUTP nick-end labeling (TUNEL) reaction mixture at 37°C for 60 min. After washing the sections 3 times with PBS, cell nuclei were visualized after staining with 4',6-diamidino-2-phenylindole (DAPI; cat. no. C1002; Beyotime Institute of Biotechnology, Shanghai, China) in the dark. Finally, sections were analyzed and images were captured under a BX53 fluorescence microscope (magnification, ×400, Olympus Corporation). The apoptotic index (apoptotic cells/total cells × 100%) was calculated from five random fields per section with ImageJ software version 1.46r (National Institutes of Health, Bethesda, MD, USA).

### Western blotting

Kidney tissues were homogenized in RIPA lysis buffer (cat. no. P0013B; Beyotime Institute of Biotechnology). Nuclear proteins were extracted using a Nuclear and Cytoplasmic Protein Extraction Kit (cat. no. KGP150; NanJing KeyGen Biotech Co., Ltd., Nanjing, China) according to the manufacturer′s protocol. The proteins were quantified using the bicinchoninic acid method. A total of 40 μg protein per sample was separated by 10% sodium dodecyl sulfate-polyacrylamide gel electrophoresis and then transferred to polyvinylidene fluoride membranes. The membranes were blocked with 5% skimmed milk at room temperature for 2 h. Subsequently, membranes were incubated overnight at 4°C with primary antibodies against nuclear factor (NF)-erythroid 2-related factor 2 (Nrf2; cat. no. ab62352; 1:2000), NF-κB p65 (cat. no. ab16502; 1:2000), heme oxygenase-1 (HO-1; cat. no. ab68477; 1:10,000; all Abcam, Cambridge, UK), caspase-3 (cat. no. 19677–1-AP; 1:800), Toll-like receptor 4 (TLR4; cat. no. 19811–1-AP; 1:800), B-cell lymphoma-2 (Bcl-2; cat. no. 12789–1-AP; 1:2000), Bcl-2-associated X protein (Bax; cat. no. 50599–2-IG; 1:5000; all Proteintech Group, Inc., Rosemont, IL, USA), cleaved caspase-3 (cat. no. 9664; 1:1000; Cell Signaling Technology, Danvers, MA, USA), lamin B (cat. no. BA1228; 1:800), and β-actin (cat. no. BM0627; 1:800; Boster Biological Technology, Wuhan, China), followed by incubation with secondary goat-anti-mouse (cat. no. BA1051) or goat-anti-rabbit (cat. no. BA1054) horseradish peroxidase-conjugated antibody (both 1:50,000; Boster Biological Technology) at room temperature for 2 h. Bands were visualized using enhanced chemiluminescence reagents (Thermo Fisher Scientific), and band intensities were detected with Glyko BandScan gel analysis software (Glyko, Novato, CA, USA).

### Statistical analysis

Results are expressed as mean ± standard deviation (SD). One or two-way analysis of variance was used to determine significant differences. Differences between groups were analyzed using Tukey’s multiple comparisons tests and considered significant at *p* < .05. All statistical tests were conducted with GraphPad Prism software version 7.0 (GraphPad Software, Inc., San Diego, CA, USA).

## Results

### Diabetes aggravates I/R-induced kidney injury

**Figure 1.**
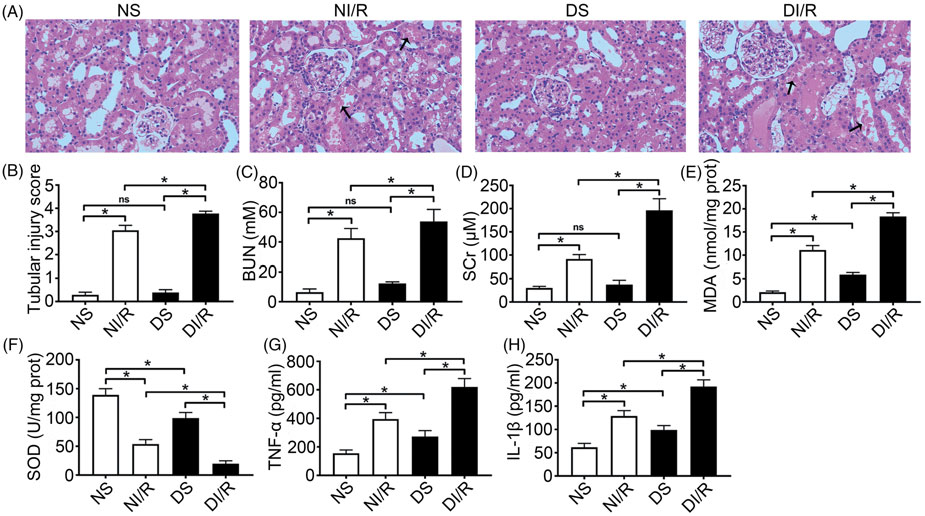
Diabetes aggravates renal ischemia and reperfusion injury. (A) Representative H&E images of renal tissues (magnification, ×400), arrows indicate tubular necrosis. (B) Histopathological scoring. (C) BUN concentration. (D) SCr concentration. (E) MDA content in kidney tissues. (F) SOD activity in kidney tissues. (G) TNF-α level in kidney tissues. (H) IL-1β level in kidney tissues. The results are presented as mean ± SD. *p < .05.

To investigate the effect of hyperglycemia on susceptibility to RI/RI, we established an STZ-induced type 1 diabetes model. As shown in Table 1, 8 weeks after STZ treatment, we confirmed that hyperglycemia, weight loss, polydipsia, and polyphagia were significantly greater in diabetic rats compared to normal rats. Renal tissue morphology is shown in Figure 1(A,B). No abnormalities were detected in tubular cells in the DS and NS groups (*p* > .05). However, following I/R, kidneys in the diabetic group exhibited severe tubular damage in the proximal tubules, including tubular dilatation, loss of the brush border, cast formation, and cell lysis (*p* < .05). The trends of serum BUN and SCr levels were consistent with histological findings (Figure 1(C,D)), indicating more serious RI/RI following hyperglycemia.

**Table 1.** General characteristics of rats 8 weeks after STZ injection.

	NS (*n* = 8)	NI/R (*n* = 8)	DS (*n* = 8)	DI/R (*n* = 8)
Random blood glucose (mM)	6.5 ± 0.7	5.8 ± 0.8	23.2 ± 2.3*	24.0 ± 2.9*
Body weight (g)	437.8 ± 15.6	444.1 ± 17.2	311.8 ± 12.9*	319.4 ± 14.8*
Water intake (ml/kg/day)	106.6 ± 4.5	105.9 ± 3.4	760.3 ± 12.1*	763.8 ± 11.5*
Food consumption (g/kg/day)	69.5 ± 2.9	67.4 ± 3.7	182.1 ± 6.0*	180.8 ± 7.2*

The results are presented as mean ± SD.

*p < .05 versus NS group and NI/R group.

### Diabetes aggravates I/R-induced oxidative stress, inflammation, and renal cell apoptosis

As shown in Figures 1(E,F), we observed a significant increase of MDA content together with a significant reduction in SOD activity in diabetic rats, which might indicate that hyperglycemia could induce chronic oxidative stress. Moreover, hyperglycemia further exacerbated oxidative abnormalities after I/R compared to the NI/R group (*p* < .05). Levels of the pro-inflammatory cytokines TNF-α and IL-1β were also higher in diabetic rats compared to normal rats, and I/R induced further increases (Figure 1(G,H), *p* < .05).

**Figure 2.**
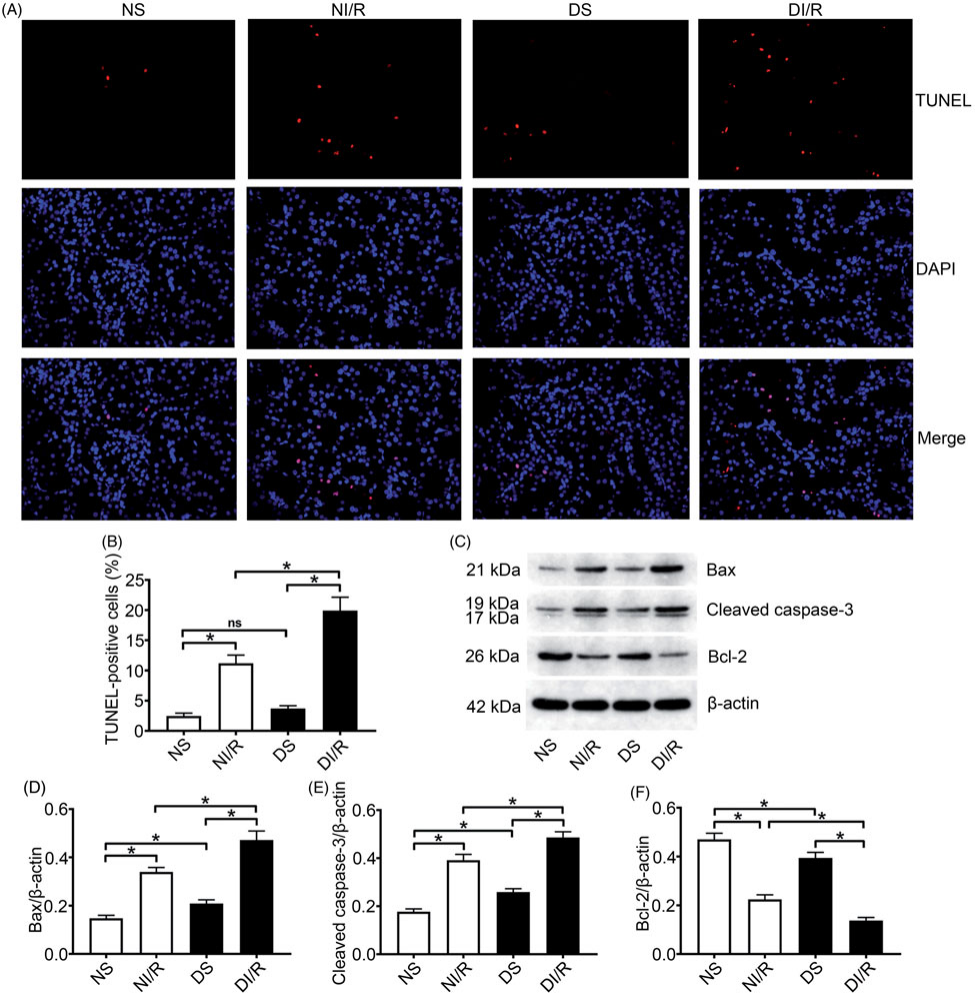
Diabetes promotes I/R-induced apoptosis of renal tubular epithelial cells. (A and B) Renal apoptosis was detected by TUNEL assay. (A) Representative fluorescence microscopy images (magnification, ×400) and (B) quantitative analysis for TUNEL staining. (C–F) The expression of apoptosis-related proteins was examined by western blotting. (C) Representative blots and quantitative analysis of western blotting for (D) Bax, (E) cleaved caspase-3, and (F) Bcl-2. The results are presented as mean ± SD. *p < .05.

TUNEL results were evaluated to estimate apoptosis in renal tubular epithelial cells (Figure 2(A,B)). The number of TUNEL-positive (apoptotic) cells obviously increased after I/R in both normal and diabetic rats, whereas the most apoptotic cells were found in the DI/R group (*p* < .05). Apoptosis-related protein expression in kidney tissue was examined by western blotting (Figure 2(C–F)). Bax and cleaved caspase-3 levels significantly increased after I/R in both the normal and diabetic groups, whereas the largest increases (for both proteins) occurred in the DI/R group (*p* < .05). Bcl-2 expression in both the NI/R and DI/R groups was markedly decreased compared with that in their corresponding sham groups, whereas decreased Bcl-2 expression was most pronounced in the DI/R group (*p* < .05).

### Diabetes blocks the Nrf2/HO-1 pathway and amplifies TLR4/NF-κB signaling after I/R stimulation

**Figure 3.**
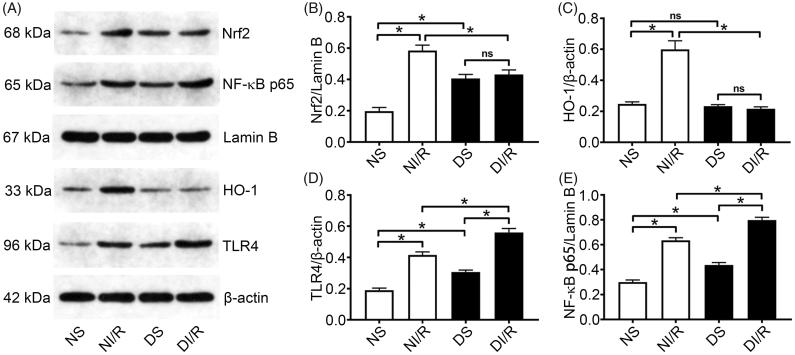
Diabetes blocks the Nrf2/HO-1 pathway and amplifies NF-κB signaling after I/R. (A) Representative western blotting images and quantification of protein levels of (B) Nrf2, (C) HO-1, (D) TLR4, and (E) NF-κB. The results are presented as mean ± SD. *p < .05.

Nrf2 is an important redox-sensitive transcription factor that mainly induces the expression of antioxidant proteins including HO-1 and protects cells from oxidative damage [29]. Western blotting (Figure 3(A–E)) revealed that the nuclear level of Nrf2 was significantly higher in the DS group compared to the NS group (*p* < .05). However, as a downstream oxidase of Nrf2, HO-1 expression levels were similar between the NS and DS groups. Consistently, although, HO-1 was upregulated after I/R in the normal group compared with the NS group (*p* < .05), it was unchanged in the DI/R group.

TLR4/NF-κB signaling is an important inflammatory pathway involved in I/R injury. Hyperglycemia alone, without I/R injury, caused significant increases in TLR4 and nuclear NF-κB levels compared with the NS group (*p* < .05), which clarified the consistent increases in pro-inflammatory cytokines levels. After I/R injury, TLR4 and NF-κB levels in the diabetic group were significantly increased compared to the NI/R group (*p* < .05).

### TBHQ pretreatment alleviates I/R-induced oxidative stress, inflammation, and apoptosis in diabetic rats

**Figure 4.**
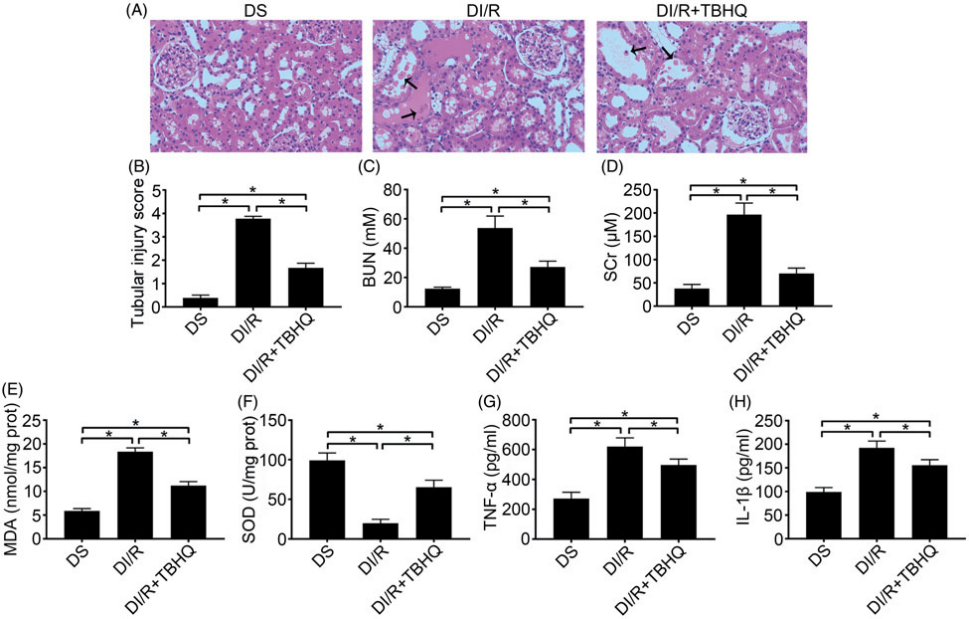
TBHQ pretreatment alleviates acute kidney injury, oxidative stress, and inflammatory responses after I/R in diabetic rats. (A) Representative images of H&E staining of renal tissues (magnification, ×400), arrows indicate tubular necrosis. (B) Histopathological scoring. (C) BUN concentration. (D) SCr concentration. (E) MDA content in kidney tissues. (F) SOD activity in kidney tissues. (G) TNF-α level in kidney tissues. (H) IL-1β level in kidney tissues. The results are presented as mean ± SD. *p < .05.

**Figure 5.**
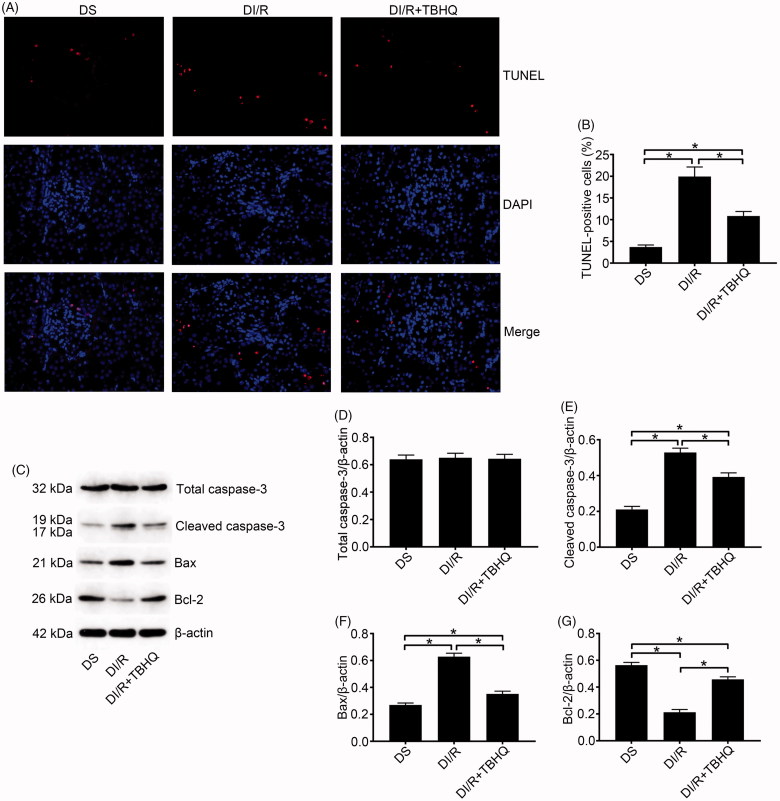
TBHQ pretreatment reduces I/R-induced apoptosis in renal tubular epithelial cells in diabetic rats. (A and B) Renal apoptosis was detected by TUNEL assay. (A) Representative fluorescence microscopy images (magnification, ×400) and (B) quantitative analysis of TUNEL staining. (C–G) The expression of apoptosis-related proteins was examined by western blotting. (C) Representative blots and quantitative analysis of western blotting for (D) total caspase-3, (E) cleaved caspase-3, (F) Bax, and (G) Bcl-2. The results are presented as mean ± SD. *p < .05.

As shown in Figure 4(A–D), TBHQ pretreatment prevented RI/RI in diabetic rats, as indicated by lower microscopic damage scores and lower serum BUN and SCr compared with the DI/R group (*p* < .05). It also alleviated oxidative abnormalities, as evidenced by significantly decreased MDA content and increased SOD activity compared with the DI/R group (Figure 4(E,F), *p* < .05). Moreover, TBHQ pretreatment lowered TNF-α and IL-1β levels compared with the DI/R group (Figure 4(G,H), *p* < .05). The effects of TBHQ on tubular apoptosis and relevant proteins in the setting of hyperglycemia are shown in Figure 5(A–G). Compared to the DI/R group, TBHQ administration before I/R markedly reduced apoptosis (*p* < .05). Consistently, TBHQ pretreatment significantly decreased the expression of pro-apoptotic proteins (Bax and cleaved caspase-3) and increased Bcl-2 expression compared with the DI/R group (*p* < .05).

### TBHQ pretreatment inhibits NF-κB signaling

**Figure 6.**
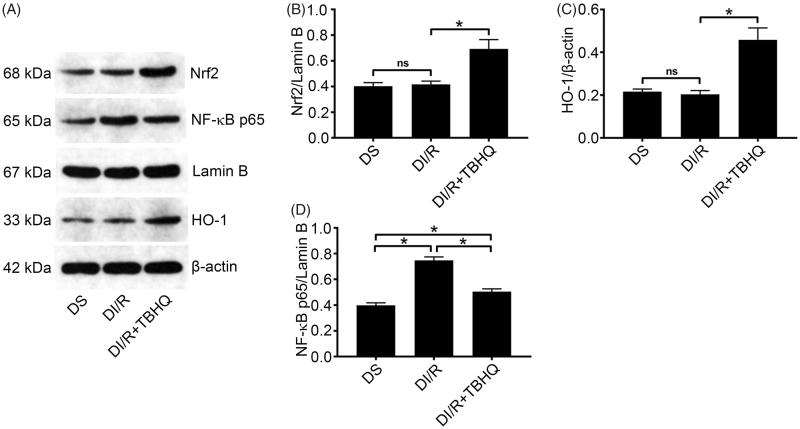
TBHQ pretreatment activates the Nrf2/HO-1 pathway and inhibits NF-κB expression after I/R in diabetic rats. (A) Representative western blotting images and quantification of protein levels of (B) Nrf2, (C) HO-1, and (D) NF-κB. The results are presented as mean ± SD. *p < .05.

As shown in Figure 6(A–D), TBHQ pretreatment obviously elevated the Nrf2 level and then significantly enhanced downstream HO-1 expression compared with the DI/R group (*p* < .05). Furthermore, TBHQ significantly attenuated the NF-κB increase in diabetic rats exposed to I/R compared with the DI/R group (*p* < .05).

## Discussion

Our findings in an STZ-induced diabetic rat model confirm that hyperglycemia exacerbates RI/RI in terms of tubular damage and increased BUN and SCr concentrations via aggravation of oxidative stress, TLR4/NF-κB-mediated inflammation, and apoptosis. Furthermore, we also demonstrate that TBHQ pretreatment can protect kidney from I/R injury by enhancing the antioxidant capacity and favorably modulating the inflammatory reaction, resulting in lower pro-inflammatory cytokine and pro-apoptotic protein expression, even in the setting of hyperglycemia.

Its complex vascular network renders renal tissue more vulnerable to I/R damage, especially in diabetes, which is more likely to lead to acute renal failure [30]. Peng et al. found no between-group (non-diabetic vs. diabetic) differences regarding the histological observations and serum BUN and SCr concentrations in the absence of I/R. However, these parameters were significantly increased after I/R injury, particularly in the diabetic group [23]. Likewise, after culturing BRL-3A hepatocytes in 5.5 or 25 mM D-glucose media for 6 to 96 h, cell viability was similar between the two groups under normoxic conditions. Following hypoxia/reoxygenation, viability was significantly decreased with 25 mM glucose incubation [9]. Consistent with this, we found no significant variation in histopathologic findings or renal function between the DS and NS groups. After I/R insult, renal function deterioration and tubular damage tended to be more serious in the DI/R group compared to the NI/R group, which indicates that the kidney is sensitive to I/R injury under hyperglycemia.

Oxidative stress refers to the imbalance between oxidant and antioxidant activity in the body; it plays an important role in regulating pathophysiological responses in kidney disease and is characterized by increased generation of ROS and decreased ROS scavenging capacity [31]. Under normal circumstances, ROS produced in metabolic processes can be cleared by endogenous antioxidant enzymes such as SOD [32], which catalyzes the decomposition of superoxide radicals into hydrogen peroxide. During I/R, an excessive amount of ROS is generated when blood flow is recovered, and these species damage ischemic tissue when they exceed the scavenging capacity. MDA, a product of lipid peroxidation, is a marker of oxidative stress to predict the degree of lipid peroxidation [33]. In diabetes, sustained hyperglycemia is the main mediator underlying increased ROS production [34], leading to chronic oxidative stress. Hyperglycemia induces higher ROS baseline levels in diabetic heart and liver tissues [9,35]. Consistent with previous studies, we observed that high glucose exposure *in vivo* increased basal renal oxidative stress, as reflected by increased MDA content and decreased SOD activity. However, it is unclear how chronic oxidative stress induced by hyperglycemia influences RI/RI. This study confirmed that preexisting renal oxidative stress in diabetic rats was further aggravated after I/R, accompanied by higher pathological scores and serum creatinine levels compared with the NI/R group. This suggests that chronic hyperglycemia-induced oxidative stress makes the kidney more vulnerable to acute I/R injury.

Inflammation is an important part of the innate immune response that protects tissues from damage and initiates their repair. However, excessive inflammatory reactions may exacerbate tissue damage. In addition to direct damage to cellular components, overproduced ROS following reperfusion can trigger a subsequent inflammatory response, which in turn aggravates I/R injury [3]. I/R-induced inflammation, so-called aseptic inflammation, is characterized by neutrophil recruitment and cytokine and chemokine productions [3]. Hyperglycemia reportedly aggravates renal I/R injury by exacerbating the inflammatory response, as indicated by significantly increased TNF-α and IL-1β levels in serum or renal tissues [24,30]. Similarly, our current study verified that persistent hyperglycemia in diabetic rats was followed by increased TNF-α and IL-1β levels within kidney tissues, leading to a state of chronic, low-grade inflammation that intensified after I/R treatment. Inflammation can also induce apoptosis, which plays a crucial role in renal I/R injury and can be activated through intrinsic or extrinsic pathways [31]. TNF-α is an important cytokine involved in systemic inflammation and is the major extrinsic mediator of apoptosis. Zhang et al. demonstrated a significantly higher number of TUNEL-positive tubular epithelial cells in diabetic rats subjected to renal I/R compared to non-diabetic I/R rats, and this was accompanied by the same trend of TNF-α expression in kidney tissue [30]. An *in vitro* study showed that 2 weeks of culturing renal tubular cells in a high glucose medium induced cell adaptation to high glucose conditions, and these conditioned cells did not show higher basal apoptosis. However, these cells were more sensitive to acute injury [23]. Consistent with those *in vitro* results, our *in vivo* study did not find elevated basal apoptosis in renal tubular cells in diabetic sham-operated rats. However, renal I/R obviously increased apoptosis, elevated Bax and cleaved caspase-3 expression, and reduced Bcl-2 levels in diabetic rats compared to normal rats. Our results suggest that hyperglycemia-induced chronic inflammation and sensitivity to tubular apoptosis is involved in RI/RI exacerbation in diabetes.

TLR4 is a transmembrane receptor that plays an important role in innate and adaptive immune responses [36]. TLR4 signaling is activated by endogenous ligands including damage-associated molecular pattern molecules (DAMPs) and high-mobility group box 1 (HMGB1), which are released from damaged or stressed tissues. I/R injury produces DAMPs and triggers the signaling pathway in renal tubules, which promotes the initial injury through NF-κB-mediated inflammation [37]. NF-κB is an essential transcription factor regulating physiological processes, inflammation and apoptosis, and it can promote the expression of pro-inflammatory cytokines such as IL-1β and TNF-α, thus facilitating inflammatory cell infiltration [38,39]. During early stages of diabetic nephropathy, TLR4/NF-κB signaling is activated via upregulation of HMGB1, in turn promoting cytokine release, inflammation, and proteinuria [40]. In our current study, we observed higher TLR4 and nuclear NF-κB levels in diabetic sham-operated rats. After I/R, the TLR4-NF-κB axis was further activated in diabetic rats compared to normal controls. Taken together, these results indicate that increased sensitivity to RI/RI in diabetic rats is associated with enhanced TLR4/NF-κB-mediated inflammatory signaling in the kidney.

Nrf2 is a basic leucine zipper transcription factor that mainly upregulates the expression of antioxidant proteins including HO-1 and prevents oxidative damage [29,41,42]. Normally, Kelch-like ECH-associated protein 1 (KEAP1) binds Nrf2 in the cytoplasm to form a complex that keeps Nrf2 at a relatively stable low level [43,44]. When a cell is exposed to oxidative stress, Nrf2 dissociates from KEAP1 and enters the nucleus, where it transactivates genes driven by the antioxidant response element (ARE), thereby exerting an antioxidant effect and restoring cell homeostasis [45]. Hence, Nrf2/ARE signaling plays an important defensive role against oxidative stress. Shi et al. reported decreased basal expressions of Nrf2 and HO-1 in kidney tissues of diabetic rats, and both were further reduced after renal I/R. Conversely, the Nrf2/HO-1 pathway was activated in non-diabetic rats undergoing I/R [46]. we found that, although, the nuclear level of Nrf2 was increased to resist hyperglycemia-induced oxidative injury in the DS group, the expression of HO-1 (downstream of Nrf2) remained unaffected. Furthermore, unlike the further elevation of Nrf2 and subsequent upregulation of HO-1 observed after I/R in normal rats, Nrf2 and HO-1 were not significantly increased following renal I/R in diabetic rats. These results imply that hyperglycemia weakens the antioxidant power of the Nrf2 pathway.

Recent evidence suggests that NF-κB and Nrf2 pathways inhibit each other at the transcriptional level [47]. In a mouse liver I/R model, Nrf2 activation negatively regulated the TLR4/NF-κB pathway and repressed innate immunity [48]. In addition, high glucose pretreatment induced nuclear translocation of NF-κB and Nrf2 in hepatocytes, while Nrf2 transcriptional activity was suppressed [9]. Considering how the Nrf2/HO-1 pathway is disrupted in the setting of diabetes, we conducted subsequent experiments to determine whether pretreatment with the Nrf2 agonist TBHQ [49] could attenuate RI/RI in diabetic rats. As expected, TBHQ pretreatment obviously alleviated renal tubular injury; improved renal function; and decreased levels of oxidative stress, pro-inflammatory cytokines, and pro-apoptotic factors. Moreover, TBHQ pretreatment increased nuclear Nrf2 and HO-1 levels but decreased nuclear NF-κB. These results indicate that Nrf2 activation dampened NF-κB signaling, relieved inflammation, reduced apoptosis, and enhanced antioxidant defenses. Therefore, we can reasonably speculate that the proportion of NF-κB and Nrf2 in the nucleus is an essential factor in determining cellular and tissue inflammatory responses and oxidative stress. Although, the precise interaction between NF-κB and Nrf2 remains to be clarified, Nrf2 may be a potential target to enhance cell protection.

There are some limitations of this study. First, we employed an animal model of type 1 diabetes, where hyperglycemia was due to insulin deficiency caused by STZ destruction of pancreatic islet β-cells. The dosage, route, and frequency of STZ administration varies broadly in the literature [50]. To increase the success rate, we chose a single intraperitoneal injection with a relatively large STZ dose (65 mg/kg), and we provided adequate diet and water for confirmed diabetic rats to prevent their death. However, type 2 diabetes accounts for more than 90% of clinical diabetes cases [51]. Since hyperglycemia is a major characteristic of diabetes, the main purpose of our study was to investigate the effect of high glucose on RI/RI. Given the intrinsic interconnection between the impact of hyperglycemia on tissue damage and protein signaling, *in vitro* studies are needed to confirm these results. In addition, as insulin is a first-line treatment for diabetic patients with uncontrolled hyperglycemia during the perioperative period, we cannot exclude its potential protective effect during RI/RI. Besides effectively lowering blood glucose, insulin can decrease the susceptibility of diabetic rats to AKI via anti-apoptotic and proliferative actions [23]. Additional studies are needed to determine whether these beneficial effects are independent of blood glucose levels, and the specific mechanism remains unclear.

In summary, we provide evidence that diabetes can exacerbate RI/RI, which is associated with oxidative stress, inflammation, and tubular apoptosis. Intensified inflammation may be attributable to TLR4-NF-κB axis activation in high glucose settings. Impaired antioxidant activity of the Nrf2 pathway may mediate RI/RI aggravation after high glucose exposure. We hypothesize complex interplay between Nrf2 and NF-κB at a transcriptional level under hyperglycemic conditions, but the exact mechanism remains to be elucidated. Antioxidant pretreatment may help alleviate RI/RI in diabetic patients.
